# Effect of Enzymatic Treatment of Different Starch Sources on the *in Vitro* Rate and Extent of Starch Digestion

**DOI:** 10.3390/ijms13010929

**Published:** 2012-01-17

**Authors:** Mirosław Marek Kasprzak, Helle Nygaard Lærke, Flemming Hofmann Larsen, Knud Erik Bach Knudsen, Sven Pedersen, Anne Skov Jørgensen

**Affiliations:** 1 Department of Animal Science, Faculty of Science and Technology, Aarhus University, P.O. Box 50, Tjele 8830, Denmark; E-Mails: HelleN.Laerke@agrsci.dk (H.N.L.); KnudErik.BachKnudsen@agrsci.dk (K.E.B.K.); 2 Department of Food Science, Faculty of Life Science, University of Copenhagen, Rolighedsvej 30, Frederiksberg C DK-1958, Denmark; E-Mail: fhl@life.ku.dk; 3 Novozymes A/S, Krogshøjvej 36, Bagsværd DK-2880, Denmark; E-Mails: SvP@novozymes.com (S.P.); anneskovj@gmail.com (A.S.J.)

**Keywords:** branching enzyme, α-1,6-linkages, wheat, waxy maize, potato

## Abstract

Gelatinized wheat, potato and waxy maize starches were treated enzymatically in order to increase the degree of branching of the amylopectin fraction and thereby change the starch degradation profile towards a higher proportion of slowly digestible starch (SDS). The materials were characterized by single-pulse ^1^H HR-MAS NMR spectroscopy and *in vitro* digestion profile according to the Englyst procedure. Using various concentrations and incubation times with branching enzyme (EC 2.4.1.18) without or with additional treatment with the hydrolytic enzymes; β-amylase (EC 3.2.1.2), α-glucosidase (EC 3.2.1.20), or amyloglucosidase (EC 3.2.1.3) the proportion of α-(1–6) linkages was increased by up to a factor of 4.1, 5 and 5.8 in waxy maize, wheat and potato starches, respectively. The proportion of SDS was significantly increased when using hydrolytic enzymes after treatment with branching enzyme but it was only for waxy maize that the proportion of α-(1–6) bonds and the *in vitro* digestion profile was significantly correlated.

## 1. Introduction

Starch is the most abundant storage carbohydrate in staple foods such as cereals, roots and tubers [1,2]. In addition, starch serves as the principal energy source in the human diet and plays a special role in glucose homeostasis [3]. Consumption of easily digestible food causes a rapid rise in blood glucose and substantial fluctuation of hormones, which places high stress on the regulatory system [4,5]. In this context it is well established that source, type and processing of starch has important implications for the glycemic response and control [6].

The rate and extent of starch digestion depends on intrinsic as well as extrinsic factors. Amylose is usually less readily digestible than amylopectin as shown in the study of Åkerberg, Liljeberg and Björck [7] where bread made from flour with a high proportion of amylose resulted in a lower rate and extent of digestion compared to a white wheat bread reference. Physical, chemical and enzymatic methods, however, may be used to modify the molecular structure of starch [8] and thereby delay its rate of digestion. Among these, enzymatic methods are of special interest as they are safer for the environment and consumers and the reaction can be more specifically controlled under mild conditions and result in fewer by-products [9]. Starch containing a higher proportion of α-(1–6) linkages can be produced using branching enzyme (EC 2.4.1.18) by itself or in combination with hydrolytic enzymes such as β-amylase (EC 3.2.1.2), α-glucosidase (EC 3.2.1.20) or amyloglucosidase (EC 3.2.1.3). A study of Takii *et al.* [10] showed that mice administered by highly branched dextrins *via* a stomach sonde resulted in a lower rate of glucose absorption compared to a glucose reference.

The main objective of the present study was to examine how the content of α-(1–6) branch points in the starch structure affected the rate and/or extent of enzymatic digestion. We produced starches using different combinations of incubation time and dose of branching enzyme on different starch sources, and studied the effect of further treatment using different hydrolytic enzymes. The effect on starch structure was studied by ^1^H high-resolution (HR) magic-angle-spinning (MAS) NMR spectroscopy which in previously analysis has been used for quantification of anomeric linkages in various starches [11]. The effect on the rate of digestion was evaluated using the *in vitro* Englyst procedure [12]. By this procedure starch was separated into sub-fractions according to the rate of digestion: rapidly digestible starch (RDS), slowly digestible starch (SDS) and resistant starch (RS).

## 2. Results and Discussion

### 2.1. Single-Pulse ^1^H HR-MAS NMR Spectroscopy

**Figure 1:**
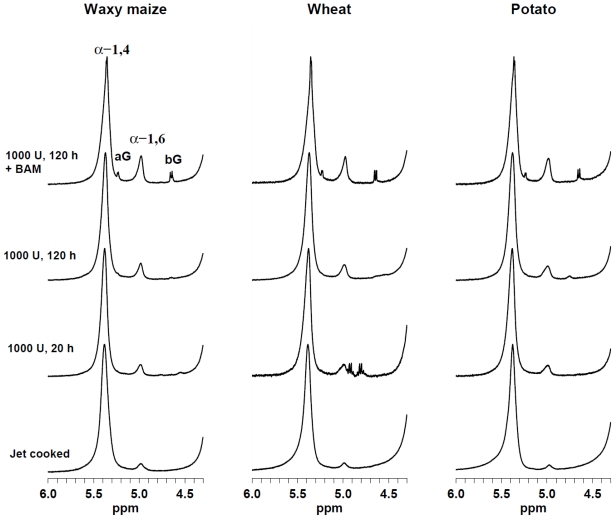
^1^H HR-MAS NMR spectra of starch gels at 85 °C. Resonances of anomeric hydrogens in α-(1–4) and α-(1–6) glucose linkages in polysaccharides as well as α-glucose (aG) and β-glucose (bG) monomer or end-group units are indicated.

The individual effect of branching enzyme (BE) and combined actions of BE and hydrolytic enzymes on the content of α-1,6-linkages in starches was examined by ^1^H HR-MAS NMR. Figure 1 shows the spectral region 4.3–6.0 ppm in the ^1^H HR-MAS NMR spectra, in which the jet cooked and selected enzymatically modified starches of the three cultivars are displayed. The two broad resonances at 5.38 and 4.98 ppm originate from the anomeric hydrogens in the α-(1–4) and α-(1–6) linkages, respectively. Furthermore, additional resonances from anomeric hydrogens in α- and β-glucose monomers or end groups at 5.24 (α) and 4.65 (β) ppm were observed in the BAM-treated starches. Relative ratios of these anomeric hydrogens are displayed in Table 1. Previously it has been shown that the degree of branching (ratio of α-(1–6) linkages to the sum of α-(1–6) and α-(1–4) linkages) was 2.8–3.4% in potato, 3.1% in wheat, and 2.3–2.7% in maize [13,14]. Our study showed that jet cooked samples resulted in 2.1%, 2.6% and 3.6% of α-(1–6) linkages in potato, wheat and waxy maize, respectively. This implies that the jet cooking process by its high pressure steam leading to the gelatinization, disruption and solubilization of the granules [15] has only slightly changed the molecular carbohydrates backbones [16]. Furthermore, there are also differences in the content of α-(1–6) branches in Glucidex consisting 2 and 6 dextrose equivalent, 4.7 *vs.* 3.3%, respectively.

Application of *Rhodothermus* amylo-(1,4→1,6)-transglycosylase (BE) induced transfer of the linear fragment of amylose (DP > 7, minimum substrate not determined) from the non-reducing end of the chain and attached it to a hydroxide oxygen on C6 in a glucose unit of the same or another chain. Hypothetically, the degree of converting amylose to amylopectin should be determined by the enzyme dose and time of incubation. In our study, using of a low dose of BE for a short time period (20 U for 20 h) only slightly increased the proportion of α-(1–6) links compared to gelatinized waxy maize material (Table 1). However, increasing the BE dose to 1000 U (1000 U 20 h) significantly increased the content of α-(1–6) bonds by 72%, 143%, and 192% compared to the jet cooked samples in waxy maize, potato and wheat starches, respectively. Prolonging the BE reaction time by 100 h (1000 U 120 h) had a marginal effect on the proportion of branches in wheat starch, whereas an increase was seen in waxy maize and potato starches. This clearly implies that the degree of polymerization of the product varies depending mainly on the dose of the branching enzyme [17] and natural variation in proportion of α-(1–6) linkages in starch sources rather than time of incubation. The hydrolytic function of BEs induces a production of linear chains which act as donors for transglycosylation [9] that configure a new compound structure. Takata *et al.* have used BE from mesophile *Bacillus cereus* to change starch into a cyclic form [18]. The studied Bacillus cereus BE catalyzed a decrease of chain length, subsequently branching and cyclization of chains and induced a second branching action on the cyclic molecules. Even though we did not analyze the conformation of the branched molecules in our experiment, we may expect that either the cyclic or high complex structure may also be obtained since we were using the BE with the similar mode of action as references.

Inclusion of β-amylase (BAM) treatment to the BE treated starch (1000 U 120 h) enhanced the number of linkages in waxy maize by 5.2%, in wheat by 5.6% and in potato by 4.7%. For waxy maize amyloglucosidase (AMG) and α-glucosidase (AGLU) were also applied to BE treated starch (1000 U 120 h) which resulted in an increase ratio of α-(1–6) linkages by 3.3% and 7.0%, respectively. Moreover it was demonstrated that AGLU was more efficient and AMG was less efficient than BAM in increasing the number of α-(1–6) linkages following BE treatment. BAM hydrolyzes α-(1–4) glucan linkages by successively removing maltose units from the non-reducing end of the chains. Consequently, BAM treated starches had 5.0%, 5.4% and 4.7% maltose in the final enzyme-treated waxy maize, wheat and potato products, respectively. AMG rapidly hydrolyzes α-(1–4) and α-(1–6) glucosidic bonds and AGLU slowly hydrolyzes *exo*-α-(1–4)-glucosidic linkages that resulted in 4.3% and 2.5% of glucose residue in AMG and AGLU material, respectively. This showed the presence of only a few by-products were left behind after purification (filtration) of the high branched starch from the low molecular weight carbohydrates.

Finally, the combined action of branching and hydrolytic enzymes led to the highest progressive formation of α-(1–6) linkages. Additionally, a higher proportion of α-(1–6) linkages found in waxy maize, was also probably due to a greater number of short branched chains present in waxy maize compared to potato and wheat, and a reduced number of long branched chains after BE treatment [19].

### 2.2. *In vitro* Digestion

The nutritional properties of jet cooked and enzymatically treated starches were evaluated by the Englyst procedure [12] (Table 2). The reference Glucidex 6 had a faster digestion rate than Glucidex 2, which is most likely due to a higher degree of hydrolysis the starch in Glucidex 6 than Glucidex 2. The raw waxy maize used for enzymatic modification in the current study contained 39% RDS, 51% SDS, and 10% RS, which was different from a previous study where it was reported to comprise of 29% RDS, 67% SDS, and 4% RS, respectively [20]. In waxy maize starch there is a large amount of short chains with a degree of polymerization (DP) of 6–12 [21]. Therefore, after gelatinization the proportions of RDS increased dramatically at the expense of SDS. Exposing the waxy maize to a low dose of BE for a short time (20 h) led to a significant increase in SDS, but not to a concurrent significant decrease in RDS. Surprisingly, in spite of a slight increase in the degree of α-(1–6) branching in waxy maize treated with a high dose of BE for 20 h, the proportion of RDS was significantly increased and the proportion of SDS significantly decreased compared to the low dose treated waxy maize. Prolongation of the incubation time to 120 h more than doubled the degree of branching compared to the untreated starch. This led to a small but significant decrease in the proportion of RDS, whereas the concomitant increase in the proportion of SDS was not significant.

The jet cooking treatment and the three major enzymatic starch modifications studied here were extended to wheat and potato starch also. A comparison between the three sources can be seen in Table 3. The native wheat starch used in the current experiment contained 47% RDS, 53% SDS, and 1% RS on total starch basis. In comparison Zhang *et al.* [22] found 40% RDS, 50% SDS and 10% RS in their starch source. The native potato starch used as the basis for enzymatic modification in the current study had a distribution of 21% RDS, 22% SDS, and 57% RS. This has a somewhat lower RS-level than our potato starch reference used in the Englyst procedure (14% RDS, 13% SDS, 73% RS) and than previously reported by Englyst *et al.* [12]. The short-term treatment with a high dosage of branching enzyme significantly increased the proportion of RDS in potato and wheat starches, and tended to decrease the proportion of SDS in potato starch. For all starches, increasing the incubation time led to a significant reduction in the proportion of RDS, while the proportion of SDS tended to increase (not significant). In spite of this, there was no change in the proportion of α-(1–6) linkages in the wheat starch, whereas the proportion increased slightly for waxy maize and potato starch. BE treatment with BAM increasing the proportion of branches, concomitantly enhanced the proportion of SDS by 6, 13 and 5% in waxy maize, wheat and potato compared to jet cooked starches, respectively. Apart from hydrolysis of α-(1–4) linkages, amyloglucosidase also release α-(1–6) bonds to produce glucose. However, the hydrolysis of α-(1–6) linkages, takes place at a slower rate than that of α-(1–4) bonds [19,23]. Therefore, a greater number of α-(1–6) linkages in produced starches limited the hydrolysis rate in the *in vitro* digestion assay.

Regarding the proportion of RS in the starches a similar trend was seen; in wheat and potato short term incubation with BE alone, or long-term BE treatment plus additional BAM treatment, led to a lower proportion of RS than jet cooking or long-term BE treatment alone. While long-term BE treatment alone led to a reduced proportion of RS compared to its jet-cooked counterpart in potato, starch treated for a long time with addition of BAM resulted in a significantly lower content of RS in both wheat and potato compared to their jet-cooked counterparts. Ao *et al.* [19] suggested that enzymatic branching not only increases the amount of α-(1–6) branches and shortens chains, but also leads to a new structure with more exposed long interior chains that might retrograde and thereby reduce the enzyme susceptibility.

The effect of using different hydrolytic enzymes after the high-dose long term treatment with BE was investigated on the waxy maize (Table 4). Addition of AGLU after the BE treatment led to the largest reduction in proportion of RDS and simultaneously increased the proportion of SDS more than any other treatment. Hence, this preparation also had the highest content of α-(1–6) branches, indicating that a major change in degree of branching is necessary in order to have an impact on the *in vitro* digestion rate. Addition of BAM and AMG did not have a similar effect and was not significantly different from the BE treated waxy maize starch concerning the proportion of RDS rate, and led only to insignificant increases in SDS. Therefore, the selection of hydrolytic enzyme after branching treatment had significant meaning.

In the present investigation, there was significant reduction in the total starch content for all enzymatic treatments compared to the non-enzymatic treated starch. A similar study [19] has shown that branching resulted in by-products such as isomaltose, isomaltotriose and panose. However, apart from the previously mentioned maltose found in BAM treated starches, our analysis showed only trace amounts of maltotriose and maltotetraose in a few starches, and a complete lack of isomaltotriose and panose in the products. Measurement of nitrogen showed that the protein content was below 1.5% in all starches. Furthermore, the level of ash was below 1% with the exception of the jet cooked potato starch, which had a content of 3.4%. In summary, BE decreased the content of total starch, and additional treatment by hydrolytic enzymes further reduced the total starch level. This tendency was more pronounced for BAM than for AMG and AGLU.

### 2.3. Relation Between Degree of Branching and *in vitro* Digestion Rate

The relationship between the proportion of α-(1–6) linkages and RDS, SDS, RS was investigated. In waxy maize starch, RDS decreased and SDS increased in parallel with a higher proportion of α-(1–6)-linkages; the correlations for RDS was *r* = 0.85 (*p* = 0.0153), and for SDS *r* = 0.87 (*p* = 0.0111). For wheat and potato starches the same trends were seen but the correlations were not significant (*p* > 0.05) probably because the comparisons were based on only a few samples. Across starch sources and treatments, the results also indicate trends for decreasing proportion of RDS and increasing proportion of SDS, but with much weaker correlations (RDS *r* = 0.49, *p* = 0.0447 and SDS *r* = 0.76, *p* = 0.0004).

Le *et al.* [9] have shown the combined effect of amylo-(1,4–1,6)-transglycosylase (BE) and Bacillus stearothermophilus maltogenic amylase (BSMA) on tapioca starch which resulted in 9.7% of extra branch linkages and significantly lowered α-amylase affinity, turnover and efficiency. Furthermore, another study [21] has shown the combined effect of β-amylase- and transglucosidase-treated maize starch which resulted in 13% of α-(1–6)-linkages, significantly decreased RDS content and significantly increased SDS proportion compared to reference, indicating 61% RDS, 34% SDS and 5% RS of TS. Presently, BE + BAM treated wheat starch and BE + AGLU treated waxy maize starch resulted in 35% and 33% SDS, respectively, whereas the content of α-(1–6)-linkages was 13.2% and 15.3%, respectively. For comparison the BE + BAM treated waxy maize starch contained 13.4% α-(1–6)-linkages but only 31% SDS. Overall, the results indicate that enzymatic modification of starch changed the degree of branching but the impact on *in vitro* digestion varies, which may be attributed to starch source [9], enzyme sources [24], dose, time of incubation and other processing conditions e.g., the storage temperature [25].

## 3. Experimental Section

### 3.1. Preparation of Enzymatically Modified Starches

Commercially available wheat-, potato- (KMC, Denmark) and waxy maize starches (Cargill Nordic A/S, Denmark) were used as substrates for enzymatic treatment. The general procedure for enzymatic treatment was as follows; starch slurry (10% DM) was gelatinized by jet cooking at pH 6.1 and 140 °C with a holding time of 5–7 min. After cooling to 75 °C, 20 or 1000 U/g DM *Rhodothermus Obamensis,* amylo-(1,4→1,6)-transglycosylase (BE, EC 2.4.1.18) was added and incubated for 20–120 h. The enzymatic reaction was stopped by reducing pH to 3.5, increase the temperature to 93 °C and maintain it for 30 min. Afterwards pH was adjusted to 5.5, the reaction mixture was filtered, evaporated and spray dried. In three cases, an additional step was performed for waxy maize in which addition of hydrolytic enzymes was included; pH was adjusted to 5.5, 4.5 or 5.0 and the mixtures were incubated with either β-amylase (BAM, EC 3.2.1.2) for 24 h, α-glucosidase (AGLU, EC 3.2.1.20) for 23 h with previous inhibition of branching enzyme, and amyloglucosidase (AMG, EC 3.2.1.3) for 2 h at 60 °C without inactivation of branching enzyme. The enzymes were inactivated by decreasing pH to 3.5, increasing the temperature to 93 °C and maintain it for 30 min. Subsequent handling was performed as for the samples only treated with BE except that an ultrafiltration step was included prior to the evaporation step in order to remove most of the low molecular weight sugars formed by the enzymatic hydrolysis. For reference, starch samples were heat treated without addition of enzyme at pH 6.1 in 75 °C for 20 h, hereafter pH was decreased to 3.5 and the mixture incubated at 93 °C for 30 min, and treated as the samples with BE. Two samples of commercially available Glucidex 2 and 6 containing maltodextrins with 2 and 6 percent of dehydrated glucose syrup (Roquette, France) were used as reference samples. An overview of treatments is shown in Table 5.

### 3.2. Single-Pulse ^1^H HR-MAS NMR Measurement

Samples for ^1^H HR-MAS NMR analysis were prepared by mixing 0.8–1.6 mg of starch powder with 55 μL D_2_O containing 5.8 mM Trimethylsilyl-2,2,3,3-tetra deuteropropionic acid (TSP-*d*_4_).

All experiments were carried out on a Bruker Avance 400 NMR spectrometer (BrukerBioSpin, Rheinstetten, Germany) operating at a Larmor frequency of 400.13 MHz for ^1^H, using a double-tuned HR-MAS probe equipped with 4-mm (o.d.) rotors. Single-pulse ^1^H NMR spectra were acquired using a recycle delay of 5 s, a spectral width of 8278 Hz, an acquisition time of 1.98 s, 64 scans and a spin-rate of 14 kHz. In order to ensure gelatinization of the starches the experiments were carried out at 85 °C.

All spectra were apodized by Lorentzian line broadening of 0.3 Hz prior to Fourier transformation and referenced to TSP-*d*_4_ at 0.0 ppm. Quantification of the anomeric α-(1–4) and of α-(1–6) hydrogens was performed by integration of the spectral regions 5.25–5.55 ppm and 4.9–5.1 ppm, respectively.

### 3.3. *In vitro* Starch Digestion

The samples were characterized with respect to rate and extent of digestion into RDS, SDS and RS according to the *in vitro* Englyst procedure [12] with minor modifications. All samples were analyzed in triplicate using raw potato starch as reference, which was analyzed in duplicate. Dry matter content of the samples was determined by drying to constant weight at 105 °C for 20 h. For the *in vitro* digestion assay, 600 mg of spray dried samples were mixed with 50 mg guar gum (Sigma Aldrich, St. Lois, MO, USA) in 50 mL centrifuge tubes with caps. Five glass beads were added to each tube. The samples were treated with 5 mg/mL of pepsin A (EC 232-629-3, P7000, 460 U/mg solid, Sigma Aldrich, St. Lois, MO, USA) in 0.05 M HCl in a shaking bath water (160 strokes/min) at 37 °C for 30 min. pH was changed to neutral by addition of 10 mL 0.25 M sodium acetate buffer. Afterwards, 5 mL of an enzyme mixture containing pancreatin 0.13 g/mL (E.C. 232-468-9, P7545, Sigma Aldrich, St. Lois, MO, USA), invertase 0.29 mL (EC 3.2.1.26, 857 EU/mL, BDH Chemicals), amyloglucosidase 0.15 mL (E.C. 3.2.1.3, 540 U/mL, Megazyme International Ireland Ltd., Wicklow, Ireland), was added at one min. intervals. After 20 and 120 min of incubation 500 μL was transferred to a tube containing 35 mL 66% ethanol and centrifuged. The remainder was gelatinized in a boiling water bath for 30 min. Subsequently the tubes were placed at room temperature to cool down. After cooling, 10 mL of 7 M KOH was added to the tubes and incubated for 30 min. One mL of the content was transferred to a tube containing 10 mL 0.5 M acetic acid and 200 μL of 80 U/mL amyloglucosidase solution (E.C.3.2.1.3 Megazyme International Ireland Ltd., Wicklow, Ireland) was added. The tubes were placed in a water bath at 70 °C for 30 min followed by a boiling water bath for 10 min. After cooling to room temperature 35 mL of milli-Q water was added and the tubes were centrifuged. Measurement of glucose was performed in duplicate by transferring 25 μL sample into a micro test plate and adding 260 μL GODPOD (Megazyme, Ireland). The plate was incubated at 40 °C for 20 min. and the absorbance measured spectrophotometrically (UV-VIS Spectrophotometer, Shimadzu) at 510 nm.

### 3.4. HPAEC-PAD Measurement

The starches were analyzed for their content of mono- and oligosaccharides. 50 mg sample was mixed with 10 mL 50% EtOH (v/v) containing 250 mg/L arabinose as internal standard and incubated at 65 °C for 60 min with occasional mixing (3 times). After centrifugation at 2000 × g for 10 min, 0.25 mL of the liquid phase was diluted 20 times with water, and filtered with a 22 μm nylon filter. The oligosaccharides were analyzed by high pressure anion exchange chromatography with pulsed amperometric detection (HPAEC-PAD) (Dionex, Sunnyvale, CA, USA). Separation of the carbohydrates was performed with a Dionex CarboPac PA-100 column (250 mm × 4 mm) equipped with a CarboPac PA-100 guard column (50 mm × 4). The carbohydrates were eluted by two different gradients prepared from water (eluent A), 0.225 M NaOH (eluent B), and 0.5 M NaOAc (eluent C). For determination of glucose, maltose and maltotriose, the flow was also 1 mL/min, and eluent B changed from 80% to 65% at 11 min, then to 60% at 13 min, and returning to 80% at 17 min and kept there for 8 min. Simultaneously eluent C was changed from 15% to 30% at 11 min, increasing to 35% at 13 min, and then returned to the initial concentration at 17 min. A long elution program with a flow rate of 0.7 mL/min was used for determination of maltotetrose, panose and isomaltotriose. In this procedure eluent B changed from 7.5% to 11.5% at 17 min, was kept constant until 10 min, then increasing to 25% at 27 min and kept constant until 57 min, where it increased to 53% and was kept there until 69 min, where it was returned to the initial 7.5% and kept until 80 min. Eluent C was added at 1% at 27 min, changed to 20% at 57 min, then aborted at 69 min. Quantification of the carbohydrates was carried by an external standard using mixtures in concentration ranging from 2–20 mg/L.

### 3.5. Calculation and Statistical Analysis

The values for total starch (TS), RDS, SDS and RS were calculated from the values of released glucose after 20 min (G_20_), 120 min (G_120_), FG (free glucose) and TG (total glucose) according to the method of Englyst *et al.* [14]:

$$\begin{array}{c} \text{TS}=(\text{TG}-\text{FG})\times 0.9\\ \text{RDS}=({\text{G}}_{20}-\text{FG})\times 0.9\\ \text{SDS}=({\text{G}}_{120}-{\text{G}}_{20})\times 0.9,\;\text{and}\\ \text{RS}=(\text{TG}-{\text{G}}_{120})\times 0.9 \end{array}$$

where, the factor 0.9 denote the conversion from monosaccharide to polysaccharides. TS is obtained after KOH hydrolysis which can result in loss of glucose, and TS could, consequently, be lower than G_120_. Therefore, RS can be negative in some materials.

Statistical analysis was performed using SAS for Windows, version 9.2 (SAS, 2007) by the use of three following different variance models:

Samples of waxy maize treated with different concentrations of BE at different time together with the untreated starch and two reference starches (Glucidex 2, Glucidex 6) were first analyzed by one-way Analysis of Variance (ANOVA).

The effect of starch source and treatment for the samples treated with 1000 U BE for 20 and 120 h, 1000 U 120 h + BAM, and untreated samples were followingly analyzed according to a two-way ANOVA:

$$\begin{array}{c} Y_{\text{ijk}}=\mu +\alpha_{\text{i}}+\beta_{\text{j}}+\alpha \beta_{\text{ij}}+{ɛ}_{\text{ijk}}\\ i=1,\;2,\;3;j=1,\;2,\;3,\;4,\;5;\;k=1,2,3. \end{array}$$

where *Y*_ijk_ is the dependent variable, *μ* the overall mean, *α*_i_ the effect of source, *β*_j_ is the effect of treatment, *αβ*_ij_ the interaction between source and treatment, and *ɛ*_ijk_ the error.

Waxy maize samples that had been treated with BE for 120 h either alone or in combination with either BAM, AGLU, or AMG was analyzed together with the untreated waxy maize in a one-way ANOVA.

Finally, the relationships between the proportion of α-(1–6) linkages and rates of digestion (RDS and SDS) were tested by regression model:

$$Y_{\text{i}}=\alpha +\beta x_{\text{i}}+{ɛ}_{\text{i}}$$

where *α* is the intercept on the *Y*-axis, *β* is the slope and *ɛ*_i_ is a random normally distributed variable [26]. Data are presented as least square means with their standard errors (SE). Differences were considered to be significantly different when *p* < 0.05.

## 4. Conclusions

By branching enzymes and additional hydrolytic enzymes, it is possible to enhance the degree of α-(1–6) branching of wheat, potato and waxy maize starches. At a high level of branching, the *in vitro* rate of digestion shifted towards more SDS and less RDS in waxy maize, wheat and potato starches. Assessing waxy maize starch sources, a significant relation was found between the degree of branching and the *in vitro* digestion pattern. However, even the double increase of branching ratio by individual BE treatment was insufficient to profoundly change the profile of digestion in all of the starches.

## Figures and Tables

**Table 1:** Relative content (%) (±0.3%) of α-glucose, β-glucose monomers (or end-groups) and α-1,6 and α-1,4 branching in wheat, potato and waxy maize jet cooked and enzymatic modified starches.

Source	α-glucose	β-glucose	α-1,6	α-1,4	α-1,6/(α-1,6 + α-1,4)
Waxy maize					
Jet cooked			3.6	96.4	3.6
20 U 20 h			4.9	95.1	4.9
1000 U 20 h			6.2	93.8	6.2
1000 U 120 h			7.9	91.9	7.9
1000 U 120 h + BAM	0.4	1.9	13.1	84.6	13.4
1000 U 120 h + AMG	0.5	2	11.2	86.4	11.5
1000 U 120 h + AGLU	0.5	1.6	14.9	83	15.3
Wheat					
Jet cooked			2.6	97.4	2.6
1000 U 20 h			7.6	92.4	7.6
1000 U 120 h			7.3	92.7	7.3
1000 U 120 h + BAM	0.3	2	12.9	84.8	13.2
Potato					
Jet cooked			2.1	97.9	2.1
1000 U 20 h			5.1	94.9	5.1
1000 U 120 h			7.5	92.5	7.5
1000 U 120 + BAM	0.5	1.7	12.2	85.6	12.5
Glucidex 2	0.6	1.8	4.6	93	4.7
Glucidex 6	1.1	2.4	3.2	93.3	3.3

**Table 2:** The effect of branching enzyme at different dosages and incubation of time on *in vitro* starch digestion rate of waxy maize starch.

	TS		RDS/TS		SDS/TS		RS/TS	
		
	% of d.m. *		% of TS (total starch)					
Jet cooked	98	a	74	bc	25	b	2	ab
20 U 20 h	96	bc	72	cd	29	a	−1	b
1000 U 20 h	95	c	76	a	25	b	−1	b
1000 U 120 h	95	bc	69	d	27	ab	4	a
Glucidex 2	95	bc	70	d	29	a	1	ab
Glucidex 6	97	ab	74	ab	25	b	0	b
SE	0.6		0.8		0.9		0.9	
*p* value	0.0065		0.0003		0.0217		0.0268	

* d.m. is dry matter. Values on the same column, followed by different letters, are significantly different (*p* < 0.05).

**Table 3:** Effect of branching enzyme at different incubation of time and addition of β-amylase on different starch sources.

	TS		RDS		SDS		RS	
		
	% of d.m.*		% of TS					
Waxy maize								
Jetcooked	98	a	74	cd	25	cd	2	cd
1000 U 20 h	95	bcd	76	bc	25	cd	−1	d
1000 U 120 h	95	bc	69	ef	27	c	4	bc
1000 U 120 h + BAM	90	fg	69	efg	31	b	1	cd
Wheat								
Jetcooked	97	ab	70	ef	22	d	8	a
1000 U 20 h	92	ef	76	b	22	d	2	cd
1000 U 120 h	94	bcd	69	efg	25	cd	6	ab
1000 U 120 h + BAM	85	h	67	g	35	a	−2	d
Potato								
Jet cooked	93	cde	67	fg	25	cd	8	a
1000 U20 h	90	f	79	a	22	d	−1	d
1000 U 120 h	93	de	73	d	24	d	4	bc
1000 U 120 h + BAM	88	g	70	e	30	b	0	d
SE	0.8		0.9		1.1		1.2	
Source	<0001		0.0169		0.1164		0.0372	
Treatment	<0001		<0001		<0001		<0001	
Source × Treatment	0.0435		0.0002		0.0344		0.0151	

* d.m. is dry matter. Values on the same column, followed by different letters, are significantly different (*p* < 0.05).

**Table 4:** The effect of application of the additional branching enzymes on waxy maize.

	TS		RDS		SDS		RS
		
	% of d.m.*		% of TS				
Jet cooked	98	a	74	a	25	c	2
1000 U 120 h	95	b	69	b	27	bc	4
1000 U 120 h + BAM	90	d	69	b	31	ab	1
1000 U 120 h + AMG	93	c	69	b	29	abc	2
1000 U 120 h + AGLU	93	c	64	c	33	a	3
SE	0.5		0.7		1.5		1.1
*p* value	<0.0001		<0.0001		0.0201		0.3588

* d.m. is dry matter. Values on the same column, followed by different letters, are significantly different (*p* < 0.05).

**Table 5:** Overview of experimental treatments.

	Waxy maize	Wheat	Potato
No enzyme	x	x	x
20 U BE, 20 h	x		
1000 U BE, 20 h	x	x	x
1000 U BE, 120 h	x	x	x
1000 U BE, 120 h + BAM, 24 h	x	x	x
1000 U BE, 120 h + AGLU, 23 h	x		
1000 U BE, 120 h + AMG, 2 h	x		

BE, branching enzyme; BAM, β-amylase; AGLU, α-glucosidase; AMG, amyloglycosidase.
