# Kasprzak, M.M., *et al.*, Correction: Effect of Enzymatic Treatment of Different Starch Sources on the *in Vitro* Rate and Extent of Starch Digestion. *Int. J. Mol. Sci.* 2012, *13*, 929–942.

**DOI:** 10.3390/ijms131217292

**Published:** 2012-12-18

**Authors:** Mirosław Marek Kasprzak, Helle Nygaard Lærke, Flemming Hofmann Larsen, Knud Erik Bach Knudsen, Sven Pedersen, Anne Skov Jørgensen

**Affiliations:** 1 Department of Animal Science, Faculty of Science and Technology, Aarhus University, P.O. Box 50, 8830 Tjele, Denmark; E-Mails: HelleN.Laerke@agrsci.dk (H.N.L.); KnudErik.BachKnudsen@agrsci.dk (K.E.B.K.); 2 Department of Food Science, Faculty of Life Science, University of Copenhagen, Rolighedsvej 30, DK-1958 Frederiksberg C, Denmark; E-Mail: fhl@life.ku.dk; 3 Novozymes A/S, Krogshøjvej 36, DK-2880 Bagsværd, Denmark; E-Mails: SvP@novozymes.com (S.P.); anneskovj@gmail.com (A.S.J.)

The authors wish to change the description of preparation of samples at Experimental Section on their paper published in *IJMS*[1].

Section 3.1

The gelatinization of samples for branching enzyme treatment was not processed by jet cooking at pH 6.1 and 140 °C with a holding time of 5–7 min, but by the adjustment of pH to 6.1 at room temperature, heated to 75 °C, adjusted to pH = 6.1, kept at 75 °C for 20 h, adjusted pH to 3.5, incubated at 93 °C for 30 min and finally adjusted pH to 5.5 at 93 °C.

Moreover, throughout the paper text and in Tables and Figures the term “jet cooked” should be replaced with “gelatinized”. The authors and publisher apologize for the inconvenience.

The corrected version can be accessed at: http://www.mdpi.com/1422-0067/13/12/17292/s1.
